# Supplementary material for: Pericytes repair engineered defects in the basement membrane to restore barrier integrity in an in vitro model of the blood-brain barrier
Source: Mater Today Bio. 2025 Sep 26;35:102361. doi: 10.1016/j.mtbio.2025.102361 (PMC12517171; doi:10.1016/j.mtbio.2025.102361)
Supplement: Multimedia component 1 [file mmc1.docx]

Supplementary Information: Pericytes Repair Engineered Defects in the Basement Membrane to Restore Barrier Integrity in an in vitro Model of the Blood-Brain Barrier.

Michelle A. Trempel^1^, Yimei Du^1^, Louis P. Widom^2^, Emily E. Reitz^1^, Alexis M. Feidler^3^, Pelin Kasap^4^, Britta Engelhardt^4^, Thomas R. Gaborski^2^, Harris A. Gelbard^3,5,6,7^, Niccolo Terrando^8,9,10^, James L. McGrath^1^

**Table S1.** Antibodies used for immunofluorescence staining

| **Antibodies** | **Fixative** | **Clone** | **Source** | **Cat. N.** | **RRID** | **Dilution** |
| --- | --- | --- | --- | --- | --- | --- |
| Mouse Anti-Human VE-Cadherin IgG2B | 4% Paraformaldehyde | 123413 | R&D Systems | MAB9381 | AB_2260374 | 1:50 |
| Rabbit Anti-Human PDGFRβ IgG | 4% Paraformaldehyde | 28E1 | Cell Signaling Technology | 3169 | AB_2162497 | 1:100 |
| Mouse Anti-Human Collagen Type IV IgG2b, κ, Alexa Fluor 647 | live | 1042 | Invitrogen | 51-9871-82 | AB_10853027 | 1:100 |
| Mouse Anti-Human Fibronectin IgG1, Alexa Fluor 488 | live | FN-3 | Invitrogen | 53-9869-82 | AB_11040270 | 1:200 |
| Rabbit Anti-Human Laminin IgG | live | Polyclonal | Invitrogen | PA1-16730 | AB_2133633 | 1:100 |
| Goat Anti-Mouse IgG Alexa Fluor 488 | N/A | N/A | Invitrogen | A11001 | AB_2534069 | 1:200 |
| Goat Anti-Rabbit IgG Alexa Fluor 568 | N/A | N/A | Invitrogen | A11011 | AB_143157 | 1:200 |

Supplementary Section S1: Validation of the distance between laminin defects.

To more thoroughly demonstrate the micropores are causing the defects seen in the monoculture devices, beyond simply aligning defects with images of the micropores, an analysis was performed to show that the distance between these dips in laminin fluorescence matched the distance between the micropores was performed. To do this a line was drawn through the laminin image in FIJI (ImageJ) and the plot profile function was used to get the mean fluorescent intensity at each point along the line. This data was then exported to Matlab where it was plotted inversely, so that the dips in fluorescence would be the peaks, and then the findpeaks function was used to identify the peaks. The distance between the peaks was averaged to find the average distance between the defects and that distance was compared to the distance between the micropores as measured by SEM imaging.


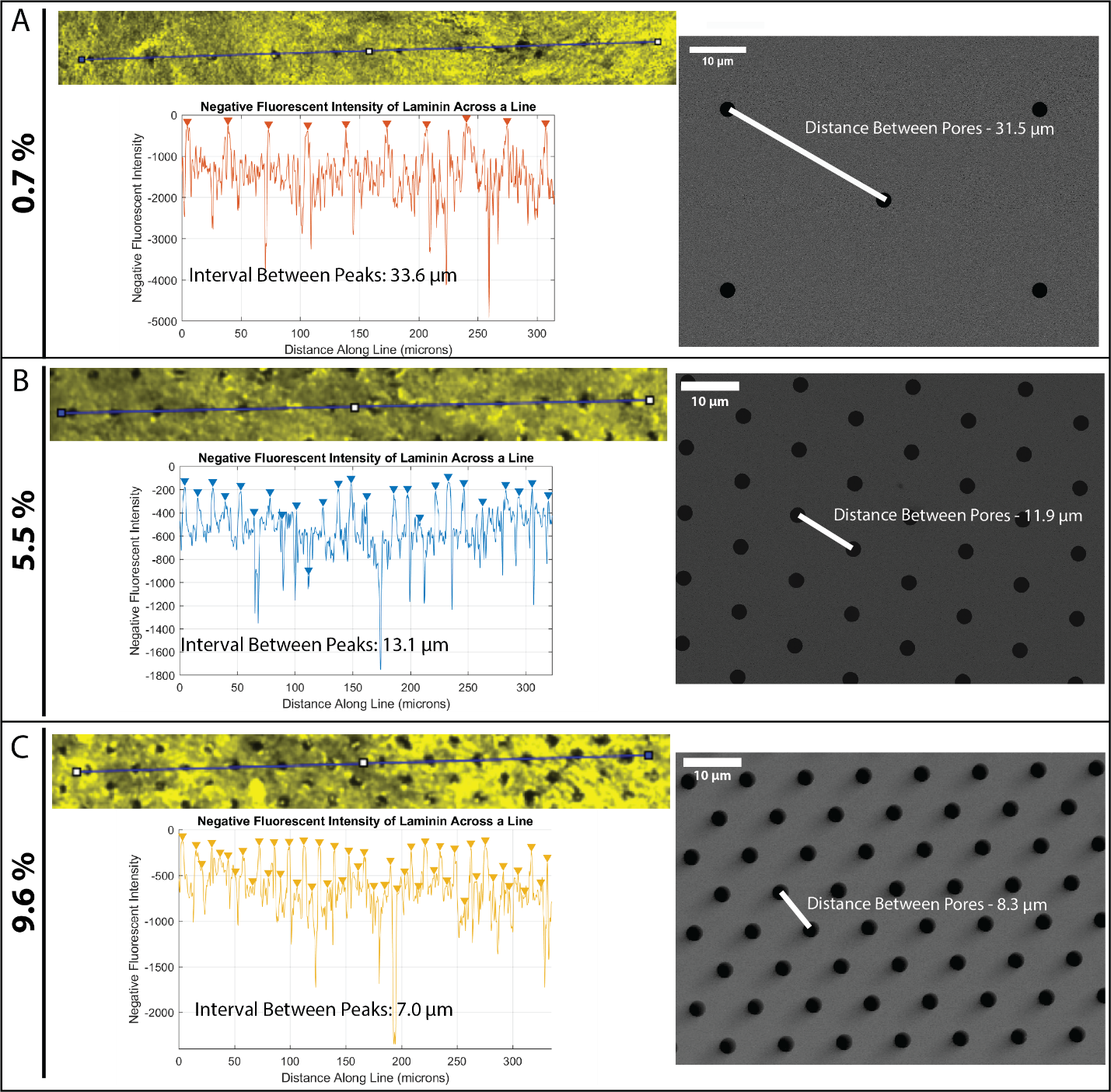


**Figure S1.** Validation of the distance between laminin defects on monoculture 3 μm DS membranes. Confocal microscopy imaging of monoculture EECM-BMECs stained for laminin (yellow) with line drawn to show where the profile data is obtained. Graph of inverted mean fluorescent intensity profile so that peaks represent dips in fluorescence with peaks identified using Matlab’s findpeaks function. The distance between these peaks was averaged to find the interval between peaks and is compared to the distance between the peaks as determined on scanning electron microscopy (SEM) images. Analysis performed on 0.7% (A), 5.5 % (B), and 9.6% (C) 3 μm DS membranes.

Supplementary Section S2: Investigation of laminin expression by BPLCs alone.

To demonstrate that the fibrous laminin we see in the coculture on dual scale membranes is a phenomenon at the interface of the coculture, and not merely a product of pericytes alone, we show representative images of laminin staining in BPLC only cultures, both on top of and underneath the membrane and deep in the BPLC layer of the cocultures, compared with EECM-BMEC-only monocultures. The laminin expression from the BPLCs is very different than the EECM-BMEC-only cultures, appearing limited to the cell body and more punctate than fibrous.


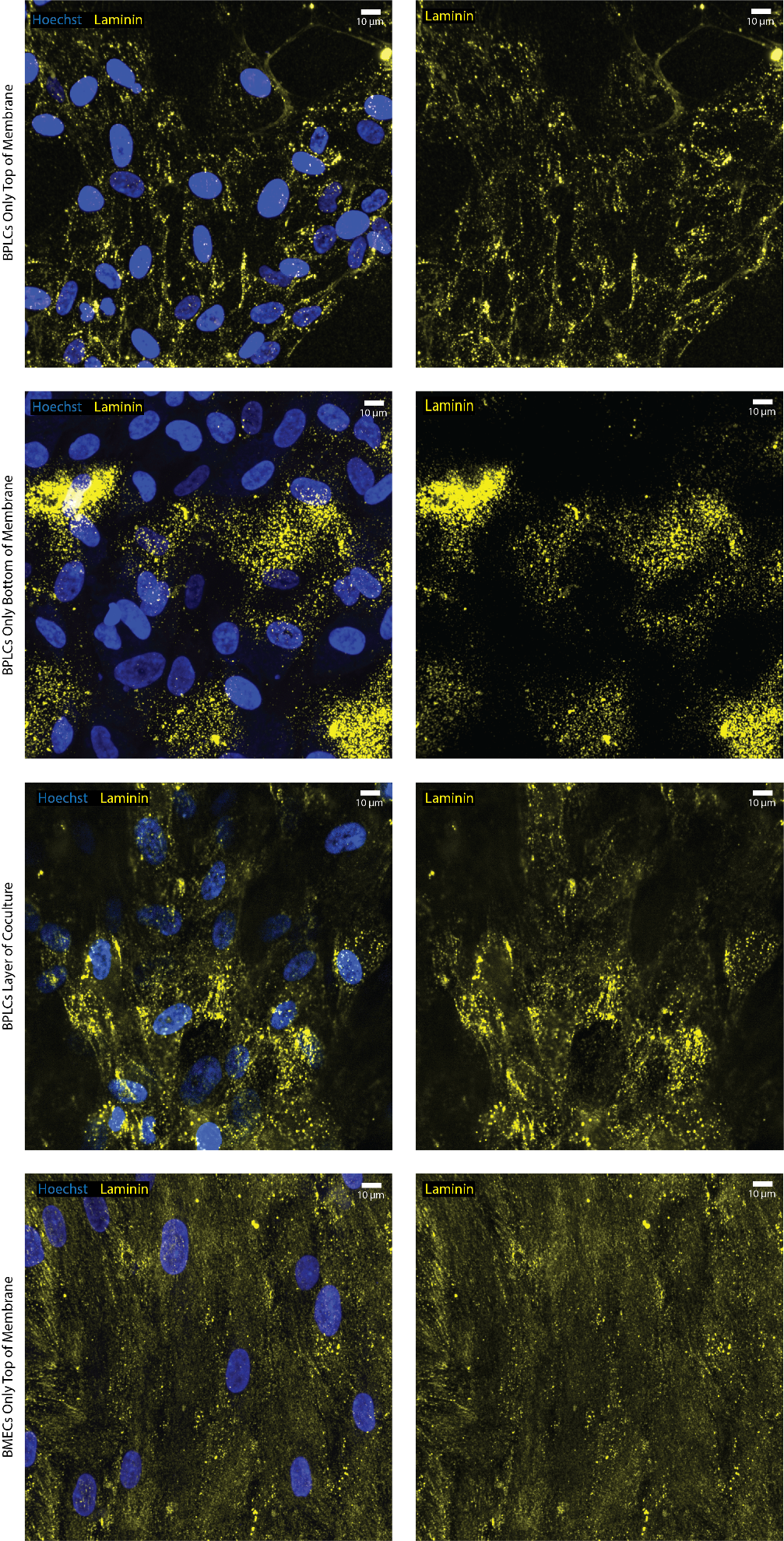


**Figure S2.** BPLCs alone produce laminin with a distinct texture as compared to EECM-BMECs. Confocal microscopy images of laminin (yellow) and Hoechst (blue) of (A) BPLCs only grown on top of the membrane, (B) BPLCs only grown beneath the membrane, (C) the BPLC layer of a coculture device and (D) EECM-BMECs only grown on top of the membrane. The BPLCs show punctate laminin expression around cells only, as opposed to more fibrous sheets laminin seen near BMECs.
